# Quantitative Analysis of Intracellular Motility Based on Optical Flow Model

**DOI:** 10.1155/2017/1848314

**Published:** 2017-07-30

**Authors:** Yali Huang, Lei Hao, Heng Li, Zhiwen Liu, Peiguang Wang

**Affiliations:** ^1^College of Electronics and Information Engineering, Hebei University, Baoding 071002, China; ^2^School of Information and Electronics, Beijing Institute of Technology, Beijing 100081, China

## Abstract

Analysis of cell mobility is a key issue for abnormality identification and classification in cell biology research. However, since cell deformation induced by various biological processes is random and cell protrusion is irregular, it is difficult to measure cell morphology and motility in microscopic images. To address this dilemma, we propose an improved variation optical flow model for quantitative analysis of intracellular motility, which not only extracts intracellular motion fields effectively but also deals with optical flow computation problem at the border by taking advantages of the formulation based on *L*_1_ and *L*_2_ norm, respectively. In the energy functional of our proposed optical flow model, the data term is in the form of *L*_2_ norm; the smoothness of the data changes with regional features through an adaptive parameter, using *L*_1_ norm near the edge of the cell and *L*_2_ norm away from the edge. We further extract histograms of oriented optical flow (HOOF) after optical flow field of intracellular motion is computed. Then distances of different HOOFs are calculated as the intracellular motion features to grade the intracellular motion. Experimental results show that the features extracted from HOOFs provide new insights into the relationship between the cell motility and the special pathological conditions.

## 1. Introduction

Cell morphology and mobility indicate physiological and pathological characters of the organism [1]. It has been demonstrated that quantitative analysis of cell morphology and mobility offers the possibility to improve our understanding of the biological processes at the cellular level [2]. Estimation of live cell mobility for analyzing dynamic properties of biological and pathological phenomena has been extensively used in clinical diagnosis and biological research, including inflammation research, drug test, wound healing, tumor genesis, and immune response [3–7]. Life information under special condition is to be uncovered by quantitative analysis of intracellular motility. However, it is difficult to measure intracellular motility due to irregular complicated cell deformation. Here, we proposed a novel approach for quantitative analysis of intracellular motility based on optical flow model.

Starting from the original work of Horn and Schunck (HS) model as well as Lucas and Kanade (LK) model [8, 9], optical flow method has been widely used in the computer vision applications for estimating the motion of the object, which is also a primary method applied in quantitative motion estimation of biological structures in light microscopy [10–12]. Vig et al. reviewed the main methods for measuring cell-scale flows, including single-particle tracking (SPT), particle image velocimetry (PIV), and optical flow [13]. Moreover, they found that although SPT and PIV techniques have been the principal means for analyzing bioflows in the cellular biophysics, optical flow technique outperforms than the formers for its relatively simple implementation and providing additional biophysical information such as local velocity [13]. Boric et al. applied optical flow method to quantify the movements of populations of cells and detect subtle cell changes in quantitative analysis of cell migration [14]. Guo et al. applied optical flow methods to track red blood cell [15]. Optical flow technique was also used in measurement of blood flow velocity in vivo [16]. All the abovementioned research work mainly regarded the cell as a whole and focus their attention on the movement of the whole cell. Little attention has been to the intracellular motion.

In this paper, we propose an improved optical flow method-based variation model for analyzing intracellular mobility in phase contrast microscopic cell images. The data term in the energy functional of the optical flow model adopts a *L*_2_ norm, which is beneficial to extract the smooth velocity field of the intracellular movement. While the smoothness term in the energy functional changes with the regional feature of the image, using *L*_2_ norm in the intracellular area and *L*_1_ norm nears the edge of the cell according to local features of the image, which is helpful to address the optical flow computation near the edge of the object. Furthermore, histograms of oriented optical flow are used to quantify the intracellular mobility.

The rest of the article is organized as follows.  Section 2 reviews related work on optical flow models.  Section 3 proposes our scheme: the improved optical flow based on variation model and characterization of intracellular motion based on HOOFs. In Section 4, we present the visualization of intracellular motion based on optical flow and apply the proposed scheme to the synthesized data and the actual data; experimental results are provided. Discussion and conclusions are given in Section 5.

## 2. The Variation Model of Optical Flow

Optical flow is defined as the vector field expressing 2-dimensional apparent motion pattern of moving object projected on the screen, and this vector field is also viewed as the velocity field of the moving object, which comprises motion and structure information of the observed object. Optical flow computation is based on the correlation in respect with time and space between the two subsequent images of a video. Although many new concepts have been proposed for dealing with different problems in optical flow models, today's optical flow is still similar to HS model or LK model [8, 9]. This kind of variation optical flow normally can be expressed as follows:

(1) $$\begin{array}{c} E\left(u,v\right)=E_{data}\left(u,v\right)+\alpha E_{smooth}\left(u,v\right), \end{array}$$

where (*u*, *v*) is the flow vector of a pixel in the 2-dimensional optical flow field; *E*_data_(*u*, *v*) is a data term and *E*_smooth_(*u*, *v*) is a smooth term, with *α* being a weight between the two terms. Normally, the data terms are composed of some constancy assumptions, such as gray value, gradient constancy assumption, Hessian conservation equations, and Laplacian conservation equations. These constancy assumptions form constraints for the solution of the variation optical flow model. Different constraints of data term have been used in different motion patterns. The smoothness term, which guarantees the existence of a unique of the optical flow model, adopts different smooth strategies in different applications. The optical flow computation based on the variation method is realized by minimizing an energy functional constituted by some data constraints and a smoothness constraint. For example, in the classical HS model, the data term adopts gray value constancy assumption and the smoothness constraint is the square of the magnitude of the gradient of the optical flow velocity. Therefore, the energy functional of HS optical flow model is expressed as follows:

(2) $$\begin{array}{c} E_{HS}\left(u,v\right)=\int_{\Omega }\left\{{\left[I_{t+1}\left(x+u\right)-I_{t}\left(x\right)\right]}^{2}+\alpha \left({\left|\nabla u\right|}^{2}+{\left|\nabla v\right|}^{2}\right)\right\}d\Omega , \end{array}$$

where *I*_*t*_(*x*) and *I*_*t*+1_(*x*) denote the successive images used to compute optical flow field; *x* = (*x*, *y*) denotes the pixel coordinate and *u* = (*u*, *v*) is the flow vector of a pixel (*u* and *v* denote the displacement of a pixel at the horizontal direction and the vertical direction, resp.); Ω indicates the image region. The optical flow field is computed by optimizing the energy functional. That is to say, *u* = (*u*, *v*) is obtained by the minimization of *E*_HS_(*u*, *v*).

In the HS optical flow model, both the data term and smooth term are in the form of *L*_2_ norm, and the implementation based on *L*_2_ norm to the image is equivalent to the isotropic diffusion. This kind of method can avoid piecewise constant in the image but lead to blurry edge and details lost. Thus, Papenberg et al. proposed a novel energy functional, in which the data term and the smooth term were expressed as $\Psi \left(s^{2}\right)=\sqrt{s^{2}+\varepsilon^{2}}$ (*ε* is a small positive number) [17]. To some extent, the minimization of Ψ(*s*^2^) results in approximate implementation based on the *L*_1_ norm. Moreover, Pock et al. proposed a total variation energy functional model, named TV − *L*1, expressed as follows [18]:

(3) $$\begin{array}{c} E_{TV-L1}\left(u,v\right)=\int_{\Omega }\left\{\left|I_{t+1}\left(x+u\right)-I_{t}\left(x\right)\right|+\alpha \left(\left|\nabla u\right|+\left|\nabla v\right|\right)\right\}d\Omega , \end{array}$$

where the data term is based on *L*_1_ norm and the smooth term adopts total variation (TV). From this energy functional, it is found that the minimization of *E*_TV−*L*1_ is equivalent to the optimization based on *L*_1_ norm. The formulation based on *L*_1_ norm to the image is equivalent to the anisotropic diffusion. This kind of implementation can preserve discontinuities near the edge in the optical flow field but result in piecewise constant in the optical flow field. In order to obtain accurate optical flow field both in the edge and in the intracellular area, we propose a flexible optical flow model functional based on the variation model, and the details are described as follows.

## 3. Methods

### 3.1. Adaptive Total Variation Optical Flow Model (Adaptive TV Optical Flow Model)

In order to compute accurately the optical flow field of the single intracellular motion both near the edge and in the intracellular area, we proposed an energy functional of optical flow model as follows:

(4) $$\begin{array}{c} E_{aTV}\left(u,v\right)=\int_{\Omega }\left\{\frac{1}{2}{\left[I_{t+1}\left(x+u\right)-I_{t}\left(x\right)\right]}^{2}+\frac{\alpha }{p}\left({\left|\nabla u\right|}^{p}+{\left|\nabla v\right|}^{p}\right)\right\}d\Omega , \end{array}$$

where *I*_*t*_(*x*) is the intensity of the pixel located at *x* = (*x*, *y*) in the frame corresponding to time *t*; *α* is a weight between the data term and the smooth term. The vector *u* = (*u*, *v*) denotes the vector of a pixel in the 2-dimension optical flow field; *u*(*x*, *y*) and *v*(*x*, *y*) are the horizontal and vertical components of the flow field. It can be concluded that *I*_*t*+1_(*x* + *u*) − *I*_*t*_(*x*) = *I*_*x*_*u* + *I*_*y*_*v* + *I*_*t*_ based on the gray value constancy assumptions and Taylor's formula [8], so we have another expression of (4).

(4‐1) $$\begin{array}{c} E_{aTV}\left(u,v\right)=\int_{\Omega }\left\{\frac{1}{2}{\left(I_{x}u+I_{y}v+I_{t}\right)}^{2}+\frac{\alpha }{p}\left({\left|\nabla u\right|}^{p}+{\left|\nabla v\right|}^{p}\right)\right\}d\Omega , \end{array}$$

where 1 < *p* < 2 is an adaptive parameter varying with the features of the image.

Our proposed adaptive TV optical flow model is to minimize the above energy functional in (4-1). That is to say, the solution of optical flow field (*u*, *v*) = arg min{*E*_aTV_(*u*, *v*)} is obtained by minimizing the energy functional (4-1). Using the calculus of the variations, the Euler-Lagrange equation for this energy functional is obtained as follows [19] (the details of solution are shown in Appendix A):

(5) $$\begin{array}{c} \begin{array}{l} \left(I_{x}u+I_{y}v+I_{t}\right)I_{x}-\alpha \cdot \nabla \cdot \frac{\nabla u}{{\left|\nabla u\right|}^{2-p}}=0\\ \left(I_{x}u+I_{y}v+I_{t}\right)I_{y}-\alpha \cdot \nabla \cdot \frac{\nabla v}{{\left|\nabla v\right|}^{2-p}}=0, \end{array} \end{array}$$

where *I*_*x*_, *I*_*y*_, *I*_*t*_ denote the partial derivatives of image brightness with respect to *x*, *y*, *t*. We choose *p* according to the local feature of the image, using the following equation:

(6) $$\begin{array}{c} p\left(x,y\right)=1+\frac{1}{1+{\left|\nabla G_{\sigma }*I\left(x,y\right)\right|}^{2}}, \end{array}$$

where *G*_*σ*_∗*I*(*x*, *y*) is the convolution of the image *I*(*x*, *y*) with the Gaussian filter *G*_*σ*_, obtaining smoother image. It is obvious that the value of |∇*G*_*σ*_∗*I*(*x*, *y*)| is high near the edge of the cell. Particularly, if |∇*G*_*σ*_∗*I*(*x*, *y*)|^2^ → ∞, then *p*(*x*, *y*) → 1. On the contrary, at the intracellular region where far away from the edge, there is |∇*G*_*σ*_∗*I*(*x*, *y*)|^2^ → 0, so we obtain *p*(*x*, *y*) → 2. In brief, the adaptive parameter *p*(*x*, *y*) is chosen so that it is smaller near a likely edge and larger away from possible edges, varying with the characteristic of the image. Therefore, the proposed adaptive TV optical flow model is equivalent to the image implementation based on *L*_1_ norm near a likely edge when *p*(*x*, *y*) ≈ 1 and based on *L*_2_ norm far away from possible edges when *p*(*x*, *y*) ≈ 2, which is helpful to deal with the problem of optical flow computation at the border.

In this study, the numerical implementation of the proposed adaptive TV optical flow model is obtained based on *p* = 1 and *p* = 2, respectively. First, if *p* = 1, we have

(7) $$\begin{array}{c} \begin{array}{l} \left(I_{x}u+I_{y}v+I_{t}\right)I_{x}-\alpha \cdot \nabla \cdot \frac{\nabla u}{\left|\nabla u\right|}=0\\ \left(I_{x}u+I_{y}v+I_{t}\right)I_{y}-\alpha \cdot \nabla \cdot \frac{\nabla v}{\left|\nabla v\right|}=0. \end{array} \end{array}$$

Note that |∇*u*| is in the denominator, in order to avoid the singularity. It is common to use a slightly perturbed norm ${\left|\nabla u\right|}_{\varepsilon }=\sqrt{{\left|\nabla u\right|}^{2}+\varepsilon }$ to replace |∇*u*|, where *ε* is a small positive number. In the same way, we used ${\left|\nabla v\right|}_{\varepsilon }=\sqrt{{\left|\nabla v\right|}^{2}+\varepsilon }$ to replace |∇*v*|. Then the gradient decent flow of (7) is

(8) $$\begin{array}{c} \begin{array}{l} u_{t}=\left(I_{x}u+I_{y}v+I_{t}\right)I_{x}-\alpha \cdot \nabla \cdot \frac{\nabla u}{\sqrt{{\left|\nabla u\right|}^{2}+\varepsilon }}\\ v_{t}=\left(I_{x}u+I_{y}v+I_{t}\right)I_{y}-\alpha \cdot \nabla \cdot \frac{\nabla v}{\sqrt{{\left|\nabla v\right|}^{2}+\varepsilon }}, \end{array} \end{array}$$

where ∇*u* = (*u*_*x*_, *u*_*y*_); ∇*v* = (*v*_*x*_, *v*_*y*_); $\left|\nabla u\right|=\sqrt{u_{x}^{2}+u_{y}^{2}}$; $\left|\nabla v\right|=\sqrt{v_{x}^{2}+v_{y}^{2}}$; ∇^2^*u* = (∂^2^*u*/∂*x*^2^) + (∂^2^*u*/∂*y*^2^); and ∇^2^*v* = (∂^2^*v*/∂*x*^2^) + (∂^2^*v*/∂*y*^2^).

To set up discrete iterative solution, using a finite difference approach on a discrete grid, our iterative solution to (8) is as follows:

(9) $$\begin{array}{c} \begin{array}{l} u^{n+1}=u^{n}+\gamma \left[\left(I_{x}u^{n}+I_{y}v^{n}+I_{t}\right)I_{x}-\alpha \cdot \nabla \cdot \frac{\nabla u^{n}}{\sqrt{{\left|\nabla u^{n}\right|}^{2}+\varepsilon }}\right]\\ v^{n+1}=v^{n}+\gamma \left[\left(I_{x}u^{n}+I_{y}v^{n}+I_{t}\right)I_{y}-\alpha \cdot \nabla \cdot \frac{\nabla v^{n}}{\sqrt{{\left|\nabla v^{n}\right|}^{2}+\varepsilon }}\right], \end{array} \end{array}$$

where *n* corresponds to discrete time and *γ* denotes the time step for each interaction. Let the indices *i*, *j*, and *k* correspond to *x*, *y*, and *t*. Here, we define some equations as follows:

(10) $$\begin{array}{c} \begin{array}{l} I_{x}\approx \frac{1}{4}\left\{I_{i,j+1,k}-I_{i,j,k}+I_{i+1,j+1,k}-I_{i+1,j,k}+I_{i,j+1,k+1}-I_{i,j,k+1}+I_{i+1,j+1,k+1}-I_{i+1,j,k+1}\right\}\\ I_{y}\approx \frac{1}{4}\left\{I_{i+1,j,k}-I_{i,j,k}+I_{i+1,j+1,k}-I_{i,j+1,k}+I_{i+1,j,k+1}-I_{i,j,k+1}+I_{i+1,j+1,k+1}-I_{i,j+1,k+1}\right\}\\ I_{t}\approx \frac{1}{4}\left\{I_{i,j,k+1}-I_{i,j,k}+I_{i+1,j,k+1}-I_{i+1,j,k}+I_{i,j+1,k+1}-I_{i,j+1,k}+I_{i+1,j+1,k+1}-I_{i+1,j+1,k}\right\}, \end{array} \end{array}$$

(11) $$\begin{array}{c} \begin{array}{l} \nabla^{2}u={\bar{u}}_{i,j,k}-u_{i,j,k}\\ \nabla^{2}v={\bar{v}}_{i,j,k}-v_{i,j,k}, \end{array} \end{array}$$

(12) $$\begin{array}{c} \begin{array}{l} {\bar{u}}_{i,j,k}=\frac{1}{6}\left\{u_{i-1,j,k}+u_{i,j+1,k}+u_{i+1,j,k}+u_{i,j-1,k}\right\}+\frac{1}{12}\left\{u_{i-1,j-1,k}+u_{i+1,j-1,k}+u_{i+1,j+1,k}+u_{i-1,j+1,k}\right\}\\ {\bar{v}}_{i,j,k}=\frac{1}{6}\left\{v_{i-1,j,k}+v_{i,j+1,k}+v_{i+1,j,k}+v_{i,j-1,k}\right\}+\frac{1}{12}\left\{v_{i-1,j-1,k}+v_{i+1,j-1,k}+v_{i+1,j+1,k}+v_{i-1,j+1,k}\right\} \end{array}. \end{array}$$

Substituting (10), (11), and (12) into (9), then we can obtain optical flow (*u*, *v*) by the iteration procedure. Second, if *p* = 2, we have

(13) $$\begin{array}{c} \begin{array}{l} \left(I_{x}u+I_{y}v+I_{t}\right)I_{x}-\alpha \cdot \nabla^{2}u=0\\ \left(I_{x}u+I_{y}v+I_{t}\right)I_{y}-\alpha \cdot \nabla^{2}v=0, \end{array} \end{array}$$

which is the same as the Euler-Lagrange equation in HS model [8]. Similarly, the iterative solution of (13) is as follows:

(14) $$\begin{array}{c} \begin{array}{l} u^{n+1}=u^{n}+\gamma \left[\left(I_{x}u^{n}+I_{y}v^{n}+I_{t}\right)I_{x}-\alpha \cdot \nabla^{2}u^{n}\right]\\ v^{n+1}=v^{n}+\gamma \left[\left(I_{x}u^{n}+I_{y}v^{n}+I_{t}\right)I_{y}-\alpha \cdot \nabla^{2}v^{n}\right]. \end{array} \end{array}$$

### 3.2. Characterization of Intracellular Motion Based on HOOF

Details on the direction and the magnitude of each pixel's velocity of intracellular motion are expressed in the optical flow field *u* = (*u*, *v*), which is a 2-dimensional vector field. However, the raw optical flow data may be of no use, as it is composed of huge volumes of data. How to obtain quantitative information from the optical flow field is always haunting researchers. A variety of techniques have been devised to address this problem. Chaudhry et al. proposed HOOF for the recognition of human actions [20]. In our study, the HOOF technique is developed to quantify intracellular motion. We perform statistical analysis to the distribution of velocity in the optical flow field. That is to say, the distribution of each pixel's velocity in the optical flow field is analyzed on the basis of statistics theory.

The feature vector of intracellular motion is extracted as follows. First, our proposed optical flow model is applied to compute optical flow fields from the successive frames of the video. Second, each flow vector in the optical flow field is binned according to its primary angle from the horizontal axis and weighed according to its magnitude. Then, we obtain the histogram of all flow vectors in the optical flow field. The function of the histogram is expressed as follows.

(15) $$\begin{array}{c} h\left(\theta_{k}\right)=s_{k},\;k=1,2,\ldots ,L, \end{array}$$

where *θ*_*k*_ = 2*π*(*k* − 1)/*L* denotes different directions (bins); *L* denotes the number of bins; and *s*_*k*_ is the sum of all velocities of each flow vector in the interval of directions [*θ*_*k*_, *θ*_*k*+1_]. The direction of each flow vector is computed as *θ* = arc tan(*v*/*u*), and the velocity of flow vector is obtained by $\sqrt{u^{2}+v^{2}}$. In our research, in order to extract the quantitative features based on the intracellular motion, the directions of flow vectors are quantified into 16, that is, *L* = 16. So the number of bins in the histogram is 16, and the height of every bin is the sum of all velocities whose angles are in the interval [*θ*_*k*_, *θ*_*k*+1_]. Lastly, the histogram is normalized. In brief, HOOFs express the features of the distribution of flow vectors in the optical flow field. Furthermore, we compute the distances of the successive HOOFs and then use these series distances as the feature vector of the intracellular mobility.

## 4. Experiments and Results

Cell microscopic images were acquired through optical phase contrast microscope at a magnification of 16,000x from the peripheral blood samples of clean healthy mice. The animal experiments were conducted by the trained staff in Beijing You'an Hospital, which is affiliated to the Capital Medical University. All the disposals are in accordance with the guideline of animal ethics. Each video lasts for 22–24 seconds, and the frame rate is 25 frames per second (every video includes 550–600 frames). It should be noted that the details of segmentation and tracking of the single cell are presented in our early work [21]. In this article, it is assumed that the cell has been segmented and tracked from microscopic images, so we analyze the intracellular motion directly.

### 4.1. Visualization of Intracellular Motion Based on Optical Flow

**Figure 1:**
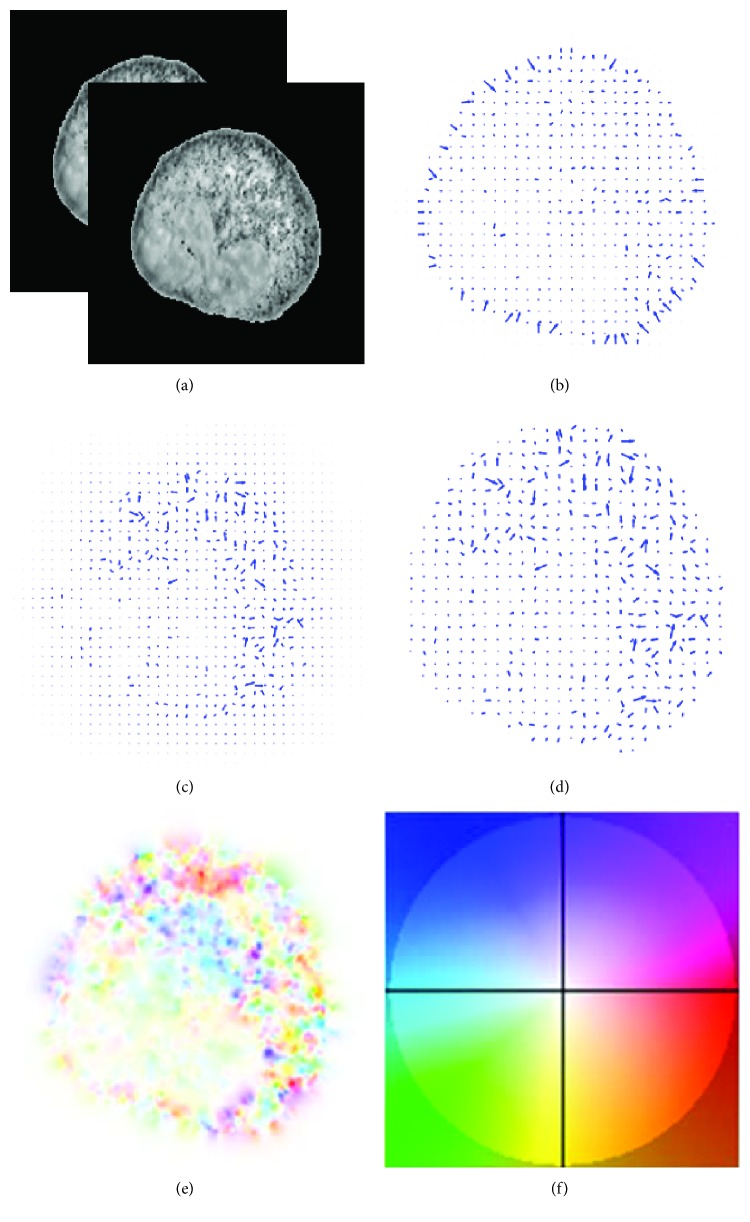
(a) The successive images from one video. (b) The optical flow field computed based on *p*(*x*, *y*) ≈ 1. (c) The optical flow field computed based on *p*(*x*, *y*) ≈ 2. (d) The optical flow field computed based on adaptive *p*(*x*, *y*) as (6). (e) Optical flow of (d) by color coding. (f) Color coding of the flow [22].

We compute optical flow field from the segmented lymphocyte in video directly, and one frame of optical flow field, which is extracted from two successive images, is shown in Figure 1. The direction of the arrow in the optical flow field denotes the direction of the intracellular motion, and the length of the arrow denotes the magnitude of the intracellular motion. In order to compare the effectiveness of the smooth term with different *p*(*x*, *y*), we compute three kinds of optical flows according to *p*(*x*, *y*) ≈ 1, *p*(*x*, *y*) ≈ 2, and adaptive *p*(*x*, *y*), as shown in Figures 1(b), 1(c), and 1(d). The results show that the formulation can preserve discontinuities by applying the robust *L*_1_ norm in the smooth term but leads to piecewise constant in the image. On the other side, using *L*_2_ norm in the smooth term can extract more details of motion but causes blurry edge. Applying the adaptive *p*(*x*, *y*) changing with the local features of the image can obtain a good balance between preserving discontinuities and optical flow details.

**Figure 2:**
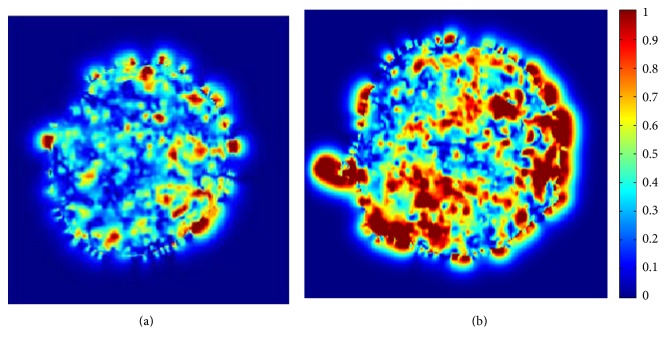
Examples of intracellular motion by color coding: red represents high velocity, while blue represents slow velocity. (a) From the NG. (b) From the AG.

In addition, we randomly chose two videos, one from the slight intracellular motion of the data group (normal group, NG), and the other is from the dramatic motion group (abnormal group, AG). Then, we computed the optical flow field of the intracellular motion and extracted the magnitude of the optical flow, as is shown by color coding in Figure 2. Based on Figure 2, it is found that the amplitude of the AG is larger than that of NG, which agrees with clinical observation.

### 4.2. HOOFs Extracted from Intracellular Motion Fields

**Figure 3:**
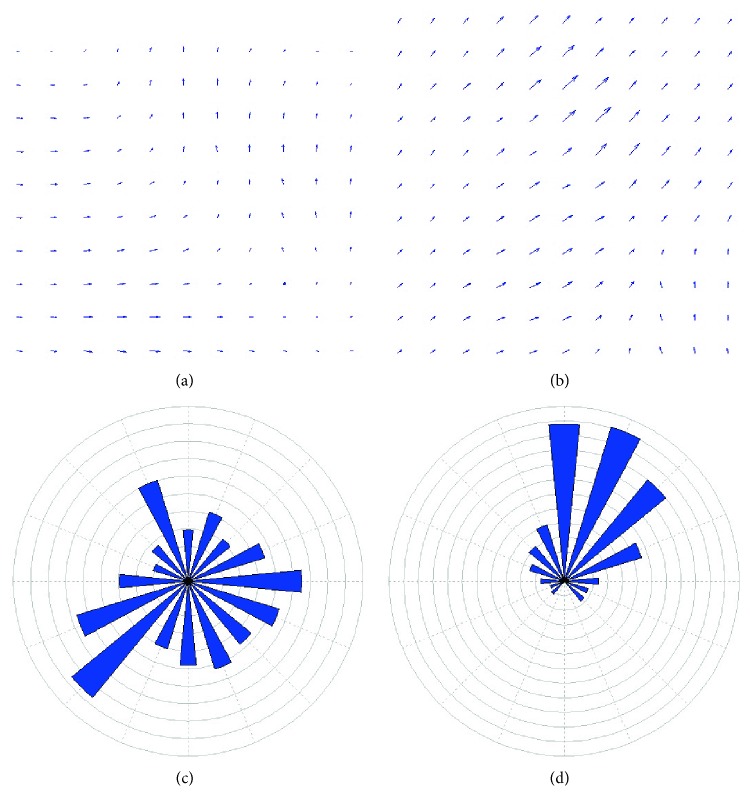
(a) The optical flow field of cell video with slight intracellular motion from NG; (b) the optical flow field of cell video with dramatic intracellular motion from AG; (c) the HOOF according to (a); and (d) the HOOF according to (b).

After the optical flow fields of the intracellular motion are computed, HOOF is extracted from optical flow fields. The characterization of the distribution of all vectors in the optical flow field is counted and expressed in HOOF, as shown in Figure 3.

From Figure 3, it is quite clear that the velocities of pixels in normal group are small and equal in each direction, while the intracellular motion in the abnormal group is more intense and the speed is large at specific direction. These HOOF results agree with the clinicians' observation: the activity of cell is enhanced in the abnormal group (when the disease occurs), that is, the cells in the abnormal group have dramatic deformation, while those in the normal group are more stable.

### 4.3. Instantaneous Velocity Extraction of Intracellular Motion Fields

To verify our proposed approach, we first verify it to the synthesized data then apply it to the actual data in Section 4.4. Four kinds of synthesized data were obtained by sampling with different frame intervals, as the following steps. Step 1: choose a frame from the cell microscopic images as the reference frame. Step 2: choose a float frame, and the interval between the float and the reference frame is 20. Step 3: a new video is obtained by sampling with the 20 frame intervals. And we name this sampled video as Celldata_20. The last step: using the similar sampling method, we obtain other three kinds of cell videos (Celldata_40, Celldata_60, and Celldata_80) with the frame interval 40, 60, and 80, respectively. In theory, the motion velocity in Celldata_20 is slower, and those in Celldata_40 and Celldata_60 are higher. Obviously, the intracellular motion in Celldata_80 is the most violent.

After four kinds of synthesized data were obtained, first, we computed the optical flow fields based on the proposed variation optical flow model of every data. Second, we calculated the mean velocity of each optical flow field as the instantaneous velocity, defined as follows:

(16) $$\begin{array}{c} V_{MeanVeloField}=\frac{1}{A_{\Omega_{1}}}\underset{\left(x,y\right)\in \Omega_{1}}{\sum }\sqrt{u^{2}\left(x,y\right)+v^{2}\left(x,y\right)}, \end{array}$$

where *u*(*x*, *y*) and *v*(*x*, *y*) are the horizontal and vertical components of the velocity at the point (*x*, *y*). *A*_*Ω*_1__ is the area of the region Ω_1_, which is a closed 2-dimensional optical flow field domain, defined as follows:

(17) $$\begin{array}{c} \Omega_{1}=\left\{\left(x,y\right):\sqrt{u^{2}\left(x,y\right)+v^{2}\left(x,y\right)}\geq thresholds\right\}, \end{array}$$

where thresholds = 0.0001 (fixed by experiments), which is used to restrict the region of the optical flow fields and restrict the optical flow data to the intracellular area.

In order to evaluate the performance of the proposed optical flow model, instantaneous velocities of four kinds of data computed based on the proposed variation optical flow model were compared with those extracted based on other two methods: the traditional HS [8] method and the Brox method [17]. All experimental parameters are set as follows. The weight parameter *α* in the energy functional is set as *α* = 15. In the Brox model, the gradient constancy assumption is removed, that is, the optimization of the data term and the smoothness term are based on *L*_1_ norm constraint approximately. In the traditional HS model, the optimization of the data term and the smoothness term is based on *L*_2_ norm constraint. In our proposed approach, the data term is based on the *L*_2_ norm constraint, while the smoothness term depends on the local region features: adopting the *L*_2_ norm in smooth area of cell and *L*_1_ norm near the edge of cell automatically. In addition, the size of Gaussian mask is 5 × 5.

**Figure 4:**
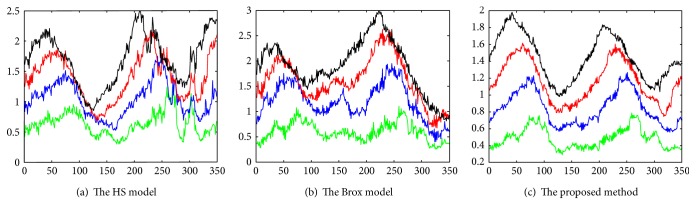
The instantaneous velocity extracted from 4 kinds of data (Celldata_20, Celldata_40, Celldata_60, and Celldata_80), by three kinds of optical flow approaches (the HS model, the Brox model, and the proposed method).

The experimental results of the abovementioned approaches (the traditional HS model, the Brox model, and our proposed model) applied to the data (four kinds of cell video: Celldata_20, Celldata_40, Celldata_60, and Celldata_80) are shown in Figure 4. The horizontal axis shows the number of frame in the image sequences; the green, blue, red, and black lines represent the instantaneous velocity calculated from the Celldata_20, Celldata_40, Celldata_60, and Celldata_80, respectively. The black lines denote the instantaneous velocity from Celldata_80, which have the highest velocity. Based on Figure 4, it is found that instantaneous velocity in the intracellular area is proportional to the frame interval. That is to say, the larger the frame interval between the float frame and the reference frame, the higher the instantaneous velocity, which agrees with the theoretical analysis. Experimental results show that our proposed adaptive optical flow model based on variation model can deal with the problems of optical flow computation at the border region to some extent and obtain optical flow values which are close to the true.

### 4.4. Feature Vector Extraction of Intracellular Motion Fields

Based on the above analysis, it can be seen that our proposed adaptive optical flow model can extract the instantaneous velocity of the synthesized data effectively. Next, we apply it to study the intracellular motion of the actual microscopic images. 120 microscopic images of cell data acquired from clean mice were used in our experiments. These data were acquired from mice with three kinds of physical state, and their corresponding intracellular motion is different: the slight motion, the moderate motion, and the dramatic motion. Every video contains more than 500 frames, which lasts for 20 seconds or so. That is to say, we analyzed the intracellular motion during a 20-second time. The frame intervals which is used for optical flow computation will influence the precision of the optical flow field. Considering that the computation cost will increase if the interframe interval is small; on the contrary, the motion characteristics cannot be captured if the interframe interval is too large, so we set the frame interval as 25 based on a large number of experiments. For example, we clip 500 frames from an original video, then sample the clipped video by 25, so a 20-frame sampled video is obtained, which will be used in the next optical flow implementation.

**Figure 5:**
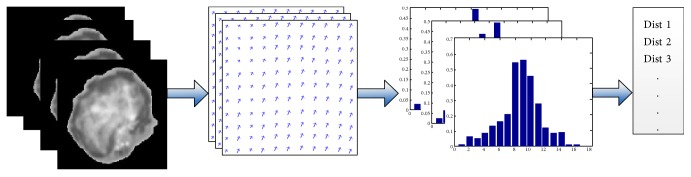
Extraction of feature vector based on intracellular motion; (a) segmented cell images; (b) successive optical flow fields; (c) histograms of optical flow (HOOFs); and (d) the Euclidean distances of HOOF (feature vector).

Afterwards, we extract the intracellular motion feature of the preprocessed data, including the following steps: first, compute the optical flow field from two successive frames of the data (the 20-frame sampled video), then we obtain 19 frame optical flow fields from one video, shown as Figure 5(b). Second, extract HOOF from every optical flow field, then we have 19 frame HOOFs, shown as Figure 5(c). Third, calculate the Euclidean distance of the two successive HOOFs, then we acquire a feature vector of intracellular motion. It is obvious that the smaller the Euclidean distances are, the slighter the intracellular motion is, and vice versa.

**Figure 6:**
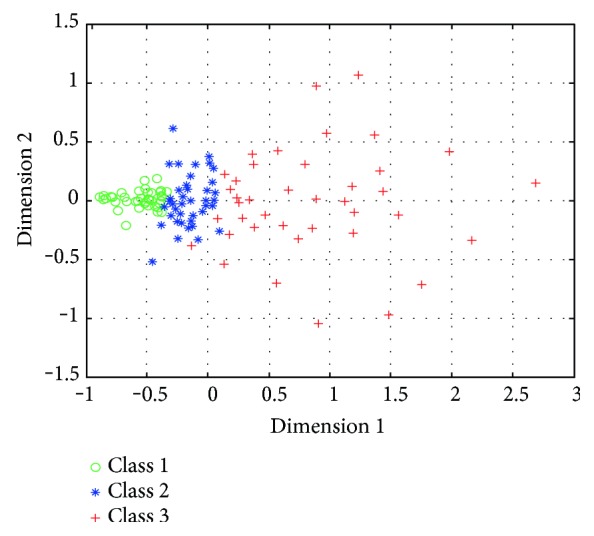
MDS projections of feature vector distances between 120 image sequences (40 from each of the three groups); the circle denotes the slight motion group, the star denotes the moderate motion group, and the plus denotes the dramatic motion group.

In order to validate the effectiveness of feature vector, we also projected the distances of the different feature vector matrix of the 120 data sets onto a two-dimensional plane using multidimensional scaling (MDS), which is a means of visualizing the level of similarity of individual cases of a dataset [23]. The result of MDS is shown in Figure 6.

Figure 6 indicates that the three groups of cell image sequence can be differentiated clearly. To further evaluate the quality of the optical feature vector, support vector machine (SVM) is used to classify intracellular motion in the microscopic images. For the data in each group, we randomly choose 20 as train sets, and another 20 for test sets. So there are 60 train sets and 60 test sets totally. The rate of classification accuracy on the test data sets by SVM achieves 91.7%.

## 5. Discussion

In this article, we proposed a novel scheme for quantitative analysis of intracellular motility based on the variation optical flow model. We applied improved variation optical flow model to visualize the velocity of intracellular motion and further coded the velocity by color, as shown in Figures 1 and 2. Moreover, HOOF was developed to quantify optical flow field of the intracellular motion, as shown in Figures 3 and 5. Lastly, we conducted two experiments to verify the proposed approach. In the first experiment, we verified the proposed approach to the synthesized data, which was obtained by sampling a microscopic cell video with different intraframe interval. Experimental results were shown in Figure 4. As shown in Figure 4(c), four kinds of velocity can be differentiated clearly based on our proposed optical flow model, while Figures 4(a) and 4(b) show that the instantaneous velocity based on HS model and Brox model cannot be distinguished effectively. The first experiment shows that the instantaneous velocity of the intracellular motion is extracted effectively by our improved optical flow model. In the second experiment, the proposed approach was applied to the microscopic cell video acquired from the clean mice. There are three kinds of cell data, representing different cases (slight intracellular motion, moderate intracellular motion, and dramatic intracellular motion) of different clean mice. Experimental results were shown in Figure 6, and it is obvious that our proposed approach can differentiate the different intracellular motion, and the rate of classification accuracy can achieve more than 90% by SVM.

The contributions of the proposed method are twofold: first, we proposed the energy functional of the optical flow model with adaptive adjustment of *p*(*x*, *y*) which change with the local feature of the image. From (6), it is obvious that *p*(*x*, *y*) = 1 near the edge of the cell, which is equivalent to *L*_1_ norm optimization for the computation of the optical flow model; therefore, the proposed model can deal with the border problem of the optical flow computation well. While *p*(*x*, *y*) = 2 in the center of the intracellular area, it is equivalent to *L*_2_ norm optimization and beneficial to extract the accurate optical flow field of the intracellular motion. Second, we calculated the HOOFs of all optical flow field extracted from the intracellular motion, and the Euclidean distances of the consecutive HOOFs were used to quantify the cell mobility in vivo.

Finally, it should be pointed out that there are still limitations in our study. First, our study was motivated by clinicians' observation that the specific cells are more active (means more morphological changes and intracellular motion) when the patient, who undergoes organ transplantation, gets the graft rejection. In consideration of security and performance, human beings with organ transplantation were replaced by the mice which undergone skin transplant in our designed scheme. Second, we just analyzed the intracellular motion in 2-dimensional space, and we will extend this study to 3-dimensional space, which agrees with the reality more.

In brief, we proposed an optical flow computation method based on variation model for quantitatively analysis of intracellular motion in microscopic images. And our research will open up new avenues for quantifying intracellular motion and better understanding of the relationship between the biological processes and the pathological phenomena at the cellular level.

## Appendix

### Derivations of the Euler-Lagrange Equation

Based on the calculus of variations, minimizing the objective functional (A.1) must satisfy the associated Euler-Lagrange (A.2).

(A.1) $$\begin{array}{c} J\left[u\left(x,y\right),v\left(x,y\right)\right]=\iint F\left(x,y,u,v,u_{x},u_{y},v_{x},v_{y}\right)dxdy, \end{array}$$

(A.2) $$\begin{array}{c} \begin{array}{l} F_{u}-\frac{\partial }{\partial x}F_{u_{x}}-\frac{\partial }{\partial y}F_{u_{y}}=0\\ F_{v}-\frac{\partial }{\partial x}F_{v_{x}}-\frac{\partial }{\partial y}F_{v_{y}}=0. \end{array} \end{array}$$

In our proposed energy functional of the adaptive TV optical flow model is as follows:

(A.3) $$\begin{array}{c} E_{aTV}\left(u,v\right)=\int_{\Omega }\left\{\frac{1}{2}{\left(I_{x}u+I_{y}v+I_{t}\right)}^{2}+\frac{\alpha }{p}\left({\left|\nabla u\right|}^{p}+{\left|\nabla v\right|}^{p}\right)\right\}d\Omega . \end{array}$$

We have that

(A.4) $$\begin{array}{c} F\left(x,y,u,v,u_{x},u_{y},v_{x},v_{y}\right)=\frac{1}{2}{\left(I_{x}u+I_{y}v+I_{t}\right)}^{2}+\frac{\alpha }{p}\left({\left|\nabla u\right|}^{p}+{\left|\nabla v\right|}^{p}\right). \end{array}$$

So

(A.5) $$\begin{array}{c} \begin{array}{l} F_{u}=\left(I_{x}u+I_{y}v+I_{t}\right)I_{x}\\ F_{u_{x}}=\alpha {\left(u_{x}^{2}+u_{y}^{2}\right)}^{\left(p-2\right)/2}u_{x}\\ F_{u_{y}}=\alpha {\left(u_{x}^{2}+u_{y}^{2}\right)}^{\left(p-2\right)/2}u_{y}, \end{array} \end{array}$$

(A.6) $$\begin{array}{c} \begin{array}{c} F_{v}=\left(I_{x}u+I_{y}v+I_{t}\right)I_{y}\\ F_{v_{x}}=\alpha {\left(v_{x}^{2}+v_{y}^{2}\right)}^{\left(p-2\right)/2}v_{x}\\ F_{v_{y}}=\alpha {\left(v_{x}^{2}+v_{y}^{2}\right)}^{\left(p-2\right)/2}v_{y.} \end{array} \end{array}$$

Substituting (A.5) and (A.6) into (A.2), then we can reach (A.7) as follows:

(A.7) $$\begin{array}{c} \begin{array}{l} F_{u}-\frac{\partial }{\partial x}F_{u_{x}}-\frac{\partial }{\partial y}F_{u_{y}}=\left(I_{x}u+I_{y}v+I_{t}\right)I_{x}-\frac{\partial }{\partial x}\left[\alpha {\left(u_{x}^{2}+u_{y}^{2}\right)}^{\left(p-2\right)/2}u_{x}\right]-\frac{\partial }{\partial y}\left[\alpha {\left(u_{x}^{2}+u_{y}^{2}\right)}^{\left(p-2\right)/2}u_{y}\right]\\ F_{u}-\frac{\partial }{\partial x}F_{u_{x}}-\frac{\partial }{\partial y}F_{u_{y}}=\left(I_{x}u+I_{y}v+I_{t}\right)I_{y}-\frac{\partial }{\partial x}\left[\alpha {\left(v_{x}^{2}+v_{y}^{2}\right)}^{\left(p-2\right)/2}v_{x}\right]-\frac{\partial }{\partial y}\left[\alpha {\left(v_{x}^{2}+v_{y}^{2}\right)}^{\left(p-2\right)/2}v_{y}\right]. \end{array} \end{array}$$

We obtain the Euler-Lagrange (A.8) of the adaptive TV optical flow model after rearranging (A.7).

(A.8) $$\begin{array}{c} \begin{array}{c} \left(I_{x}u+I_{y}v+I_{t}\right)I_{x}-\alpha \cdot \nabla \cdot \frac{\nabla u}{{\left|\nabla u\right|}^{2-p}}=0\\ \left(I_{x}u+I_{y}v+I_{t}\right)I_{y}-\alpha \cdot \nabla \cdot \frac{\nabla v}{{\left|\nabla v\right|}^{2-p}}=0. \end{array} \end{array}$$
